# Glutamic Acid Decarboxylase Concentration Changes in Response to Stress and Altered Availability of Glutamic Acid in Rabbit (*Oryctolagus cuniculus*) Brain Limbic Structures

**DOI:** 10.3390/ani11020455

**Published:** 2021-02-09

**Authors:** Izabela Szpręgiel, Danuta Wrońska, Michał Kmiecik, Sylwia Pałka, Bogdan F. Kania

**Affiliations:** 1 Department of Animal Physiology and Endocrinology, Faculty of Animal Sciences, University of Agriculture in Krakow, Al. Mickiewicza 24/28, 30-059 Kraków, Poland; rzwronsk@cyf-kr.edu.pl; 2 Department of Genetics, Animal Breeding and Ethology, Faculty of Animal Sciences, University of Agriculture in Krakow, Al. Mickiewicza 24/28, 30-059 Kraków, Poland; michal.kmiecik@urk.edu.pl (M.K.); sylwia.palka@urk.edu.pl (S.P.); 3 University Centre of Veterinary Medicine JU-AU, University of Agriculture in Kraków, Mickiewicza 24/28, 30-059 Kraków, Poland; bogdan.kania@urk.edu.pl

**Keywords:** glutamic acid decarboxylase, stress, glutamic acid, motivational brain structures

## Abstract

**Simple Summary:**

Glutamic acid decarboxylase (GAD) is an enzyme that catalyses the formation of γ-aminobutyric acid (GABA), the most important inhibitory neurotransmitter, from glutamic acid (Glu), which is the major neuromodulator in the central nervous system and is involved in most processes such as learning and memory, and in the mechanisms underlying aggressive animal behaviour. However, an excess of Glu in the neuronal space has a cytotoxic and neurodegenerative effect on neurons, and numerous studies have shown this negative effect on the proper functioning of the nervous system. GAD is therefore a key enzyme that ensures the balance between the concentration of Glu and GABA necessary for the proper functioning of brain mechanisms, including the stress response mechanism. The aim of this study was to examine if and how stress and Glu and its selected antagonists affect the level of the GAD enzyme in rabbit brain structures and, if so, in which structures these changes take place and whether GAD can be, next to adrenal hormones, an alternative marker to determine the level of stress in animals. In summary, the conducted study showed that selected rabbit brain structures showed variable GAD concentration in different ways under stressful conditions. The results presented in this paper improve our understanding of the rabbit’s limbic system and broaden our understanding of the stress response in this animal species under the influence of a stress factor. It is advisable that further studies assess precisely the Glu–GAD–GABA system under stressful conditions in other animal species, including farm animals, in particular those exposed to stress.

**Abstract:**

Glutamic acid decarboxylase (GAD) is an enzyme that catalyses the formation of γ-aminobutyric acid (GABA), the most important inhibitory neurotransmitter, from glutamic acid (Glu), which is considered the most important excitatory transmitter in the central and peripheral nervous systems. GAD is a key enzyme that provides a balance between Glu and GABA concentration. Hence, it can be assumed that if the GAD executes the synthesis of GABA from Glu, it is important in the stress response, and thus also in triggering the emotional states of the body that accompany stress. The aim of the study was to investigate the concentration of the GAD in motivational structures in the brain of the rabbit (*Oryctolagus cuniculus*) under altered homeostatic conditions caused by stress and variable availability of Glu. Summarising, the experimental results clearly showed variable concentrations of GAD in the motivational structures of the rabbit brain. The highest concentration of GAD was found in the hypothalamus, which suggests a strong effect of Glu and GABA on the activity of this brain structure. The GAD concentrations in individual experimental groups depended to a greater extent on blocking the activity of glutamate receptors than on the effects of a single stress exposure. The results obtained clearly support the possibility that a rapid change in the concentration of GAD could shift bodily responses to quickly achieve homeostasis, especially in this species. Further studies are necessary to reveal the role of the Glu–GAD–GABA system in the modulation of stress situations as well as in body homeostasis.

## 1. Introduction

The stress response is initially coordinated by several structures of the central nervous system (CNS). Following registration of the stressor by the CNS, the mammalian limbic system structures, including the hypothalamus, hippocampus, amygdala, and prefrontal cortex, are activated. During processing in the limbic system, the stressor is subjected to a “qualitative” assessment and the qualification of the stimulus as threatening results in further bodily reaction [1,2]. This response consists of the activation of two basic systems: the sympathetic–adrenal medullary system (SAM) controlled by the autonomic nervous system, and the neuroendocrine hypothalamic–pituitary–adrenal (HPA) axis. Activation of the HPA axis occurs later, as the action of the stressor, and the implications of its stimulation, can persist for a significant period, up to several days [3,4,5].

The regulation of brain motivational structures and communication between them is carried out by neurotransmitter release from neuronal presynaptic endings, which utilises glutamic acid (Glu) as a primary excitatory neurotransmitter in mammals. It constitutes the main neuromodulator for more than 50% of neurons in the CNS and participates in most of the information processing that occurs in the CNS [6]. Glu plays an important role in the process of neuronal maturation and proliferation, learning processes, and in creating memory traces and thus memory, as well as in mechanisms underlying aggressive behaviour in animals. It is also responsible for brain plasticity and is a progenitor of cell survival, as evidenced in many rodent studies [7]. In addition, Glu is responsible for the detoxification of ammonia in the brain by its binding and transport across the blood–brain barrier [8].

Glu binds to many specific protein complexes that comprise the primary types of glutamate receptors (GluRs): ionotropic (iGluRs) and metabotropic receptors (mGluRs). Within the iGluRs, three groups have been recognised: kainate receptors (KARs), α-amino-3-hydroxy-5-methyl-4-isoxazolepropionic acid receptors (AMPARs; classified as non-NMDA-type receptors), and *N*-methyl-D-aspartate receptors (NMDARs), which are ion channels that cause depolarisation of the neuronal cell membrane; their role is to control the flow of cations across the plasma membrane [9]. mGluRs, which have been identified in all brain structures, are divided into three subfamilies based on amino acid sequence homology and the similarity of intracellular signals: Group I mGluRs (mGlu1 and mGlu5), Group II mGluRs (mGlu2 and mGlu3), and Group III mGluRs (mGlu4, mGlu6, mGlu7, and mGlu8). Their central common feature is related to the activity of the transmission of intracellular signals using G proteins, which regulate the release of neurotransmitters in the CNS [10,11,12]. In mammals, including rabbits, the glutamate receptors also occur outside the CNS in many tissues, including the pituitary gland, pineal gland, adrenal glands, and sex glands, as well as on neurons of the sympathetic system, which are likely to be iGluRs [9,13].

It should be emphasised that the synthesis of the inhibitory neurotransmitter γ- aminobutyric acid (GABA) requires Glu as a substrate, as well as glutamic acid decarboxylase (GAD). GAD is not a uniformly structural enzyme, but an “isoenzyme” for which two isoforms can be distinguished: GAD67 and GAD65, occurring in mammalian neurons, including in rabbits [14,15]. Isoenzyme GAD67 is cytosolic and constantly active. It constitutes 30% of the total GAD content and is responsible for the synthesis of over 50% of GABA [16]. The isoenzyme GAD65 is located primarily in nerve endings and is associated with synaptic transmission processes [17]. Numerous studies have shown that a decrease in GAD concentration or activity leads to an increase in Glu concentration with a concomitant decrease in GABA concentration. GAD activity may be increased due to acidification or the action of weak acids [18,19,20].

In our study, we used the rabbit (*Oryctolagus cuniculus*) as an animal model to demonstrate the effect of the stressor and Glu on GAD synthesis in selected brain motivational structures. Rabbits are animals that are often used in laboratory research and in many farms where they are exposed to various stressors: physical, emotional, and others. Moreover, the rabbit is an animal species susceptible to various stress factors [21,22]. In mammals, including rabbits, the assessment of physiological response to stress can currently be characterised by measurements of glucocorticoids or catecholamines. Moreover, a significant correlation between the concentration of these hormones and brain neurotransmitters has recently been found in growing male rabbits [20]. Several previous investigations have revealed that the GABA used as a neurotransmitter plays a brain developmental function in animals [15,23,24,25]. With respect to this research topic and in relation to the welfare of smaller mammals, the results are insufficient, leaving a wide field for further research at the molecular level.

In light of the background data presented, it can be assumed that GAD, which catalases GABA synthesis from Glu, is an important enzyme involved in the stress response, primarily in the triggering of emotional states accompanying the stressful state in animals, including rabbits. The aim of this study, therefore, was to investigate GAD concentrations in the motivational structures of the rabbit brain following alteration in the organism’s homeostasis evoked by psycho-emotional stress or/and the variable availability of Glu. A better understanding of the mechanisms occurring in the limbic structures of the rabbit would allow implementation of other stress- or welfare-related parameters in farm animals and could also answer the question of how to minimise stressors in breeding, where the breeder–animal interactions are very frequent.

## 2. Material and Methods

### 2.1. Animals and Tissue Collection

**Figure 1:**
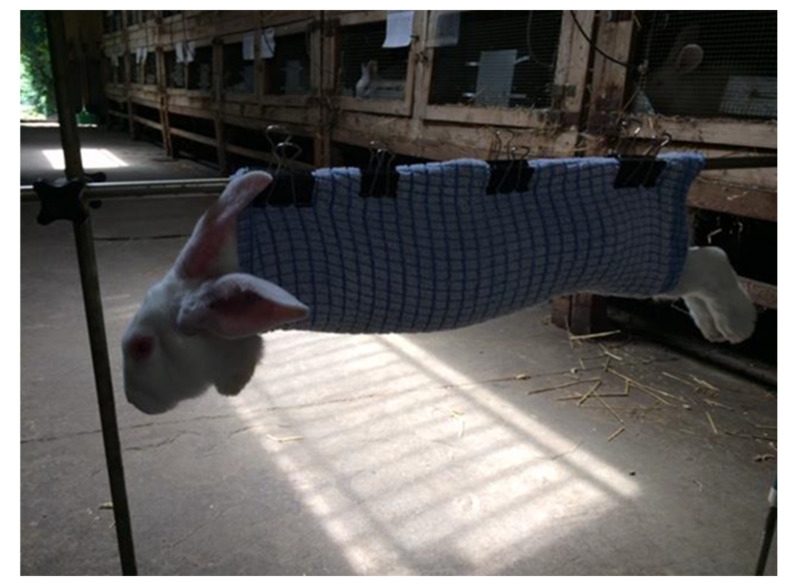
The method of inducing a stress reaction in a rabbit (photo by the authors).

The experiment was conducted on 42 Popielno White female rabbits (15 weeks of age and 2 ± 0.75 kg average bodyweight ± SEM). The animals were maintained in individual cages with dimensions consistent with the recommendations for the battery system, standing in a hall equipped with lighting (14 L:10 D), with forced and controlled ventilation and free access to water and feed (DeHeus). Before the experiment, rabbits were divided randomly into six groups (n = 7 in each group), as follows:

Group 1 (control)—Intraperitoneal injection (*i.p.*) of 2 mL of saline solution (0.9% NaCl);
Group 2 (stress)—The stress reaction was triggered by a 30 min suspension of the rabbit at a height of 40 cm above the ground in a properly prepared stand (Figure 1). This model was previously described in detail [25];
Group 3 (Glu)—Injection (*i.p.*) of Glu (G1626, Sigma-Aldrich, St. Louis, USA), dose 5.07 mg/kg b.w. (30 μM), *i.p.* in a volume of 2 mL of 0.9% NaCl;
Group 4 (Glu + stress)—Injection (*i.p.*) of Glu (as described in Group 3) and a single stressor factor (as described in Group 2);
Group 5 (Glu antagonist)—Injection (*i.p.*) of glutamate receptor antagonist (LY-341495, Sigma-Aldrich), dose 7.36 mg/kg b.w. (30 μM), *i.p*. in a volume of 2 mL of 0.9% NaCl;
Group 6 (Glu antagonist + stress)—Injection (*i.p.*) of glutamate receptor antagonist (as described in Group 5) and a single stressor factor (as described in Group 2).

Thirty minutes after the injection and/or stress exposure, rabbits from all groups were decapitated in accordance with Directive 2010/63/EU of the European Parliament and of the European Commision. The planned activities had been approved by the 2nd Local Ethics Committee at the Pharmacology Institute in Krakow (No. 116/2019).

After decapitation of each rabbit, the skin was removed from the skull cover, the meninges removed using a trepanation tool and the brain dissected. The following regions of the brain were isolated: hypothalamus, hippocampus, amygdala, and medial prefrontal cortex. Selected fragments of examined brain structures were weighed and then homogenised in liquid nitrogen. In this way, homogenates were obtained, which on the scheduled day of assay were diluted in 0.5 mL of phosphate buffer (pH = 7.5). The GAD concentration in the analysed homogenates was determined using the ready rabbit (GAD) ELISA Kit, 201-09-0310 (SunRed; Shanghai, China). The sensitivity of the method according to the manufacturer was 0.205 ng/mL and the standard curve range was 0.3–70 ng/mL at a wavelength of 450 nm. The out-of-series error of the test (CV) was <12% and the intra-series <10%. The results were converted for 1 mg of tissue.

### 2.2. Statistical Treatment of Results

The results were analysed statistically using one-way analysis of variance for repeated measurements. The significance of differences between mean values was determined by Duncan’s test. The calculations were carried out using SigmaStat 2.03 software (SPSS Science Software GmbH, Erkrath, Germany). A probability of *p* < 0.05 or *p* < 0.01 indicated statistically significant or highly statistically significant differences, respectively, between the mean values. Figures were prepared using Grapher 12 (Golden Software Inc., Golden, CO, USA).

## 3. Results

**Figure 2:**
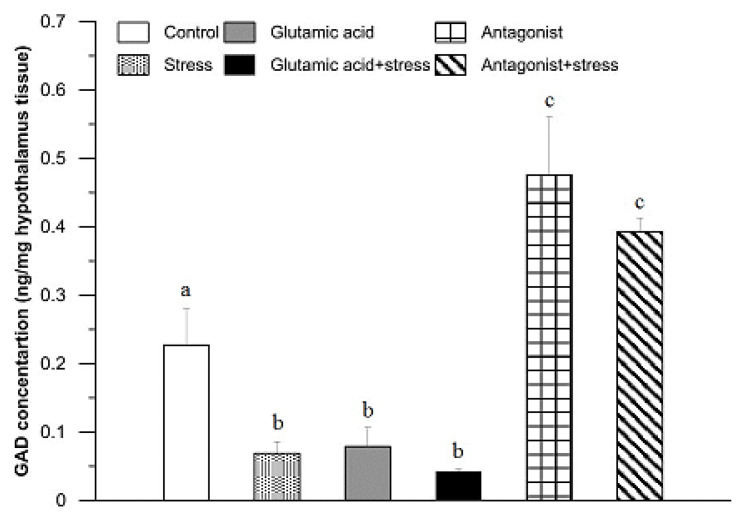
The effect of glutamic acid, stress, and glutamic acid receptor antagonist and their combinations with stress on the concentration of glutamic acid decarboxylase in the rabbit hypothalamus. Values are means ± SEM (*n* = 7). Values marked with different letters differ significantly at *p* < 0.05–0.01.

A GAD concentration of 0.23 ± 0.11 ng/mg tissue was found in the hypothalamic tissue of the control rabbits (Figure 2). In comparison with the control group, the GAD levels were significantly lower in the hypothalamic tissue of rabbits exposed to 30 min of suspension stress (0.07 ± 0.02 ng/mg; *p* < 0.01), as well as in the group of animals injected with Glu (0.08 ± 0.04 ng/mg; *p* < 0.01) and subjected to the suspension stress and those treated with Glu (0.04 ± 0.01 ng/mg; *p* < 0.01). However, after administration of the Glu receptor antagonist, a significant increase in GAD concentration was found (0.48 ± 0.14 ng/mg; *p* < 0.01) in comparison to the control group. A similar effect was noticed in the rabbits of the stressed and Glu receptor antagonist-treated group (0.39 ± 0.03 ng/mg; *p* < 0.01; Figure 2).

**Figure 3:**
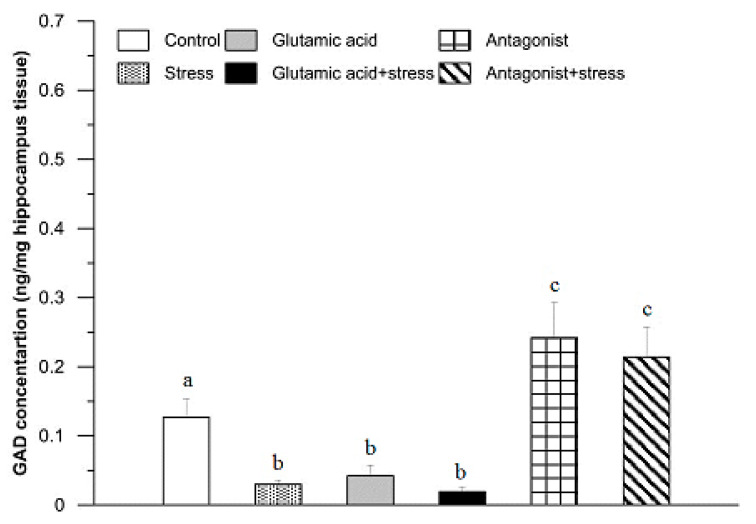
The effect of glutamic acid, stress, and glutamic acid receptor antagonist and their combinations with stress on the concentration of glutamic acid decarboxylase in the rabbit hippocampus. For further explanations, see Figure 2.

In the hippocampus of the control rabbits, the GAD concentration was 0.13 ± 0.04 ng/mg tissue (Figure 3). In comparison with the control group, the GAD levels were significantly lower after 30 min of exposure to the suspension stress (0.03 ± 0.01 ng/mg; *p* < 0.01), as well as in the group of animals injected with Glu (0.04 ± 0.02 ng/mg; *p* < 0.01) and subjected to the stress and treated with Glu (0.02 ± 0.01 ng/mg; *p* < 0.01). Exposure of the rabbits to the Glu receptor antagonist significantly elevated the GAD concentration in their hippocampus to 0.24 ± 0.11 ng/mg tissue (*p* < 0.05). A similar effect was found in the stressed and Glu receptor antagonist-treated group of rabbits (0.21 ± 0.08 ng/mg; *p* < 0.05).

**Figure 4:**
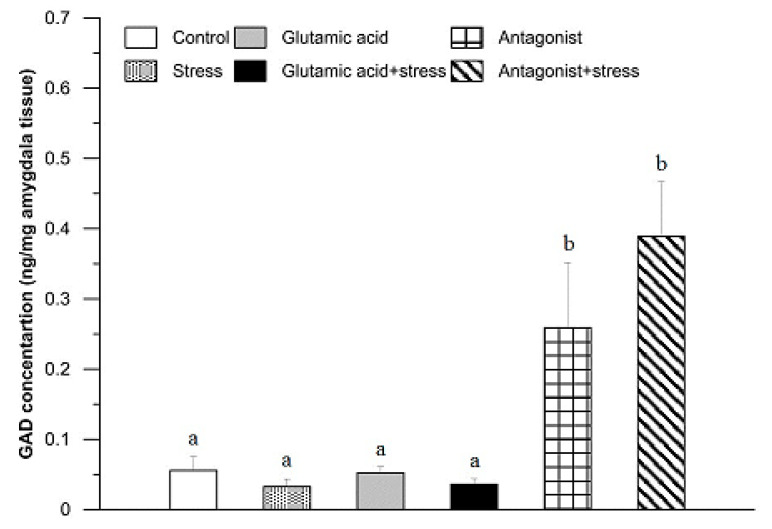
The effect of glutamic acid, stress, and glutamic acid receptor antagonist and their combinations with stress on the concentration of glutamic acid decarboxylase in the rabbit amygdala. For further explanations, see Figure 2.

The concentration of GAD in the amygdala of the control and experimental rabbits is shown in Figure 4. In comparison with the control group, where it was 0.06 ± 0.02 ng/mg tissue, there were no significant differences in GAD concentration in the amygdala of rabbits subjected to 30 min of suspension stress, injected with Glu and exposed to the stress, or treated with Glu (*p* > 0.05). On the other hand, following administration of the Glu receptor antagonist, a significant increase in GAD concentration in the amygdala was found (0.26 ± 0.16 ng/mg tissue; *p* < 0.01). A similar effect, in comparison with the control group, was noticed in the stressed and Glu receptor antagonist-treated groups (0.39 ± 0.13 ng/mg tissue; *p* < 0.01; Figure 4).

**Figure 5:**
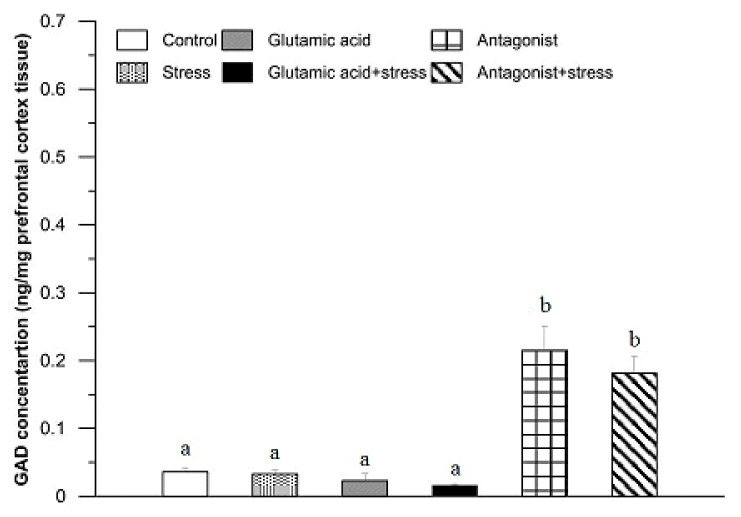
The effect of glutamic acid, stress, and glutamic acid receptor antagonist and their combinations with stress on the concentration of glutamic acid decarboxylase in the rabbit prefrontal cortex. For further explanations, see Figure 2.

A GAD concentration in the prefrontal cortex of the control rabbits was 0.04 ± 0.01 ng/mg tissue (Figure 5). There were no significant alterations in the GAD levels in the rabbits after 30 min of exposure to the suspension stress, in the group of animals injected with Glu and subjected to the stress or in those treated with Glu (*p* > 0.05). In the rabbits treated with the Glu receptor antagonist, a significant increase in GAD concentration was found (0.24 ± 0.10 ng/mg tissue; *p* < 0.01) in comparison to the control group. A similar effect was noticed in the rabbits of the stressed and Glu receptor antagonist-treated group (0.22 ± 0.07 ng/mg tissue; *p* < 0.01; Figure 5).

## 4. Discussion

The experiment described in this work clearly demonstrated a variable concentration of GAD in the motivational structures of the rabbit brain under stressful conditions and variable availability of Glu as a substrate for this enzymatic reaction. As discussed in the Introduction, GAD enables the formation of GABA from Glu in brain tissues and in the peripheral nervous system [26,27]. Numerous studies have observed that excess Glu in the interneuronal space exerts cytotoxic and neurodegenerative effects on neurons [28]. The most likely cause of excess Glu in the intercellular space is damage to glutamate transporters. It has been found that a defective glutamate transporter led to an increase in Glu concentration and, consequently, damage to motor neurons [29]. It is interesting that Glu can also be used by neurons and astrocytes as a substrate in mitochondrial metabolism to generate energy and metabolites [30]. Until recently, it was thought that neurons used only glucose as a substrate for energy and metabolite production. However, current research results contradict this view [31,32]. This suggested that the use of Glu in metabolism reduces its availability as a neurotransmitter, thereby reducing its excess and potential excitotoxicity, which may be a cellular defence mechanism against Glu excitotoxicity. The participation of Glu in metabolism may also be reflected in stress reactions. It is known that metabolism increases in times of stress, although no mechanisms have yet been discovered for the activation and regulation of a neuronal “transition” to a Glu-based metabolism [30,33]. In summary, fluctuations in Glu concentration are caused by damage to its transporters and may be caused by the variable activity of enzymes involved in its metabolism. Glu is not limited to the CNS but also acts in the peripheral nervous system, and in light of the results of many studies described above, it can be concluded that it affects brain structures by participating in a stress response and an expression of emotions. These discoveries open new and different possibilities for the modulation of Glu concentration, whose excessive accumulation has been observed in stress, especially in the context of maintaining proper homeostasis and animal welfare.

GAD is not a uniformly structural enzyme, but an “isoenzyme”, as described previously. Both isoforms are under the control of two separate genes and are regulated by different mechanisms [34,35], it is worth noting that these isoforms are the only source of GABA in the brain [36,37,38]. Isoenzyme GAD65 is located only in synaptic endings, while GAD67 is located throughout the entire cell. Their localisation indicates the role played by the GABA they produce [37,39]. GAD65 allows for the synthesis of GABA solely for the purpose of transferring information between neurons, while the GABA synthesised by GAD67 is used for purposes unrelated to neurotransmission, such as synapse formation or protection of neurons from damage [37,40]. The different functions performed by GABA are also reflected in the activity of individual isoforms. GAD67 must be constantly active, as a holoenzyme form, to ensure the proper functioning of the cell, while GAD65, an apoenzyme form, is activated only when additional neurotransmitter synthesis is needed. Under normal conditions, it is estimated that less than 50% of GAD65 remains active [41,42].

Our results indicated that among all examined brain motivational structures, the highest concentration of GAD was found in the hypothalamus of the rabbits. The hypothalamus is the main centre involved in the initiation of stress response, which is influenced by the regulation and integration of the HPA axis as well as the SAM system. It is reasonable to imagine that the high concentration of GAD found here is due to the presence of numerous and varied Glu receptors and a need to control and/or maintain the balance between Glu and GABA that is essential for the various hypothalamic functions. The results of our experiment showed that a single stressor caused significant decreases in GAD concentration, which supports the role of the hypothalamus in a stress reaction to prepare the body for a suitable response to the threat. Bowers et al. [41] conducted tests on rats subjected to severe stress. The results indicated no change in GAD65 expression and an increase in GAD67 expression in the group of rats sacrificed immediately after exposure to stress, as GAD expression levels returned to normal in the group sacrificed one hour after stressor exposure. The observed differences in relation to our research may result from different experimental conditions: among others, the duration and intensity of the stress factor, differences between species, separate determination of GAD65 and GAD67, and methods of GAD measurement, including gene expression and quantitative determination of GAD protein concentration versus ELISA. Our experiment did not allow for the assessment of changes in the concentration ratio of GAD65 and GAD67 [41]. Each neurotransmitter affects target cells through specific receptors. This mechanism is exploited where specific receptors are blocked or stimulated by exogenous compounds to target the proper functioning of the CNS and homeostasis [43]. Previous studies have focused mainly on the use of iGluR antagonists, mainly NMDARs [34,44,45,46]. For several decades, ketamine, a non-specific antagonist of NMDARs with analgesic and anaesthetic use in high doses, has been used in the clinic, especially in veterinary medicine. The properties of metabotropic receptors also suggest their beneficial regulatory potential for the stress response, but they are not currently being fully utilised [47,48,49,50]. In the present study, an mGluR antagonist was used to determine the role of mGluRs during exposure to a single stress factor. GAD concentration in the stress + mGluR antagonist group was clearly increased in the hypothalamus compared to the control group, which may imply a significant effect of these receptors on GAD secretion. However, GAD concentration did not differ significantly between the mGluR antagonist alone group and the mGluR antagonist + stress group (Figure 2). The profile of changes in GAD concentration indicated that hypothalamic mGluRs are not directly involved in the action of the stress factor, which consequently would not affect the activity of the HPA axis and the SAM system.

In analysing the hippocampus, we found that the suspension stress factor caused a decrease in GAD concentration (Figure 3). The same hippocampal effect, as seen with just the stress factor, was observed after Glu administration, as well as in the group that was exposed to the stressor after Glu injection. This suggests that mGluR, the high concentration of which we observed, among others, in the hippocampus, does not affect the activity of the GAD enzyme. It can be assumed that the stressor affected the GAD concentration by interacting with other types of receptors and that Glu diminished the course of the stress reaction. It is worth noting that the basic concentration of GAD in the hippocampus is incredibly low, which suggests that it affects a small number of Glu receptors and thus is of low importance in shaping the activity of this structure. It has been shown in rats that GAD concentration in the hippocampus increases with age [51]. In the same experiment, GABA levels were determined in young and old rats, and both groups of animals were then subjected to chronic mild stress (CMS). In older rats, a significant decrease in GAD activity and no change in Glu concentration were observed; in younger rats, the Glu concentration increased significantly. Our interpretation of this profile is that Glu lesions in older animals may have been caused by the depletion of the substrate glutamine, necessary for the synthesis of additional neurotransmitter. This is also supported by the observed decrease in GAD concentration, which was also found in our own research. GAD is responsible for the synthesis of GABA, using Glu as the main substrate, although the body has mechanisms to protect the remaining Glu against loss. In turn, in juvenile animals, a direct increase in Glu concentration is possible; thus, there is no need for the body to lower its GAD concentration. Similar studies were conducted by Pochwat and colleagues [52], and their results did not show changes in GAD67 concentration in CMS-treated rats. Additionally, Herman and Larson [53] demonstrated increased GAD65 expression in older rats and its reduction after chronic intermittent stress. The available research indicates hippocampal dysfunction in the brains of ageing animals, which can lead to serious consequences, as it is critically important for long-term memory and a centre that inhibits the activity of the HPA axis, closely related to the stress response [2].

The GAD concentrations found in the amygdala of rabbits in all groups of this study are puzzling. As a structure richly connected by neurons to other brain structures and mainly responsible for the recognition of the stressor and the activation of appropriate physiological reactions, one could expect that any change in the environment or an additional stimulus would cause changes in GAD concentration. However, in our research, the factors of stressor and Glu and their joint action did not cause significant changes in the concentration of GAD [54]. It is puzzling that the effect of the stressor, its enhancement by injection of Glu, and the effect of Glu alone did not cause any changes in the concentration of GAD. The lack of influence of Glu could be explained by the metabolic “transition” of neurons to using excess Glu as a substrate for energy and metabolite production, as described earlier. It is also interesting to observe the trend leading to increased secretion of GAD in the Glu antagonist +stress and the antagonist only groups in this structure (Figure 4). Considering both the lack of effect of Glu and increased GAD secretion in the case of the stress + mGluR antagonist group, it can be postulated that the density of mGluRs in the amygdala is lower compared to that in other structures. The previously cited study by LeWitt and colleagues [55] also showed a change in GAD67 concentration after magnesium administration. Magnesium is a natural antagonist of the NMDA receptor. This result leads to the conclusion that in this structure, it is the ionotropic receptors on which the NMDA receptor has the greatest impact in the course of the stress reaction [56,57].

The prefrontal cortex exhibited the lowest basal concentration of GAD (Figure 5). Neither Glu alone nor exposure to the stressor affected the concentration of this enzyme. It is important to note that the prefrontal cortex recognises a stressful situation, controls its course, and can slow it down by acting on the amygdala [58]. Perhaps during the experiment, the prefrontal cortex began the process of suppressing the activity of the HPA axis, and only in the case of simultaneous action of the abovementioned factors, i.e., greater stimulation, was it necessary to maintain the body’s activity. The results are consistent with a study where rats subjected to mild stress showed slight GAD67 concentration fluctuations, and the combined use of stress exposure and Mg^2+^ ions caused an increase in GAD67 concentration, which may indicate a blocking of NMDAR activity and prevention of the induction of a typical stress response [52]. This may suggest that in some regions of the brain, increased GABA levels appear together with reduced Glu level, which would confirm the results of studies by Wu and colleagues [59] on hypothalamic tissue. In turn, other studies, such as Chellappan and colleagues [25], indicate that blocking the synthesis of nitric oxide in the prefrontal cortex resulted in a significant increase in GAD concentration, which the authors described as explaining the depletion of GABA resources in the cerebral cortex. All the previously mentioned studies are especially important for rats and rabbits in the context of maintaining the welfare of these animals, due to their frequent use in laboratories.

In this study, a significant increase in GAD concentration was found in all brain structures analysed in the group of rabbits treated with the selective mGluR antagonist LY-341495 [60]. The distribution of various receptors in the brain structures is not uniform and constant, so it can be assumed that the density of one type of blocked receptor is greater than the others, and in this case, would be responsible for the increase in GAD concentration. Interpreting the results of the cited studies, it can be assumed that this increase was due to the blockade of Group I receptors, as the exclusion of activity resulted in reduced Glu secretion with a simultaneous lack of GABA secretion-inhibiting activity, which most likely led to the accumulation of GAD. However, it cannot be ruled out that the blockade of Group III receptor activity, which resulted in a disturbance of Glu and GABA metabolic pathways, caused a similar effect on these neurotransmitter secretion disturbances. The most likely blockade of Group II receptors resulted in Glu secretion and increased GAD secretion by the body to maintain Glu/GABA balance because direct injection of Glu caused a decrease in or absence of changes in GAD concentration [10,11,12]. Further research using selective antagonists for each group of glutaminergic receptors would allow for the specific determination of the impact of each on the GAD concentration in the studied motivational brain structures, as well as its mechanism of action on the organism. These studies can be used as a basis for the development of potential markers to determine the level of stress in order to improve their welfare. Further experiments should concern: determining how the concentration ratio of both GAD65 and GAD67 change as a result of a single stress factor; analysing the concentration and interrelationship between Glu and GABA; the effect of direct GAD injection and/or simultaneous stress factors; and further studies to define precisely the participation of metabotropic receptors.

In summary, the experiment showed varied GAD concentrations in the rabbit brain structures in response to a single stress factor. These results provide new information on changes in the concentration of GAD in the limbic structures of the rabbit brain following activation of the HPA axis, which may contribute to a better understanding of the mechanism of the response of these brain structures in stress situations. Our research also indicated the important role of metabotropic receptors involved in controlling GAD concentration. This enzyme can be considered essential because it is the only source of GABA in the brain and provides a balance between the two most important opposing neurotransmitters (excitatory Glu and inhibitory GABA). The results of the experiment clearly support the possibility that a rapid change in the activity of GAD may shift bodily responses to quickly achieve homeostasis. An increased understanding of the complexities underlying the impact of GAD, including its isoforms on specific brain structures, provides hope that GAD may determine the effect of the stress reaction on the body. Due to their wide application, rabbits should be thoroughly analysed, especially in terms of the limbic system. Understanding these neurological mechanisms in rabbits is also valuable because they are popular pets. Further studies are needed to precisely assess the role of the Glu–GAD–GABA system under stress conditions, primarily at the molecular level.

## 5. Conclusions

Overall, these results improve understanding of the rabbit limbic system and enhance our understanding of the stress response in this animal species under the influence of a stress factor. Moreover, these studies are only the beginning of analyses leading to the determination of animal welfare by isolating an alternative marker of potential stress levels in animals.
